# Risk messages relating to fertility and pregnancy: a media content analysis

**DOI:** 10.12688/wellcomeopenres.16744.1

**Published:** 2021-05-14

**Authors:** Olivia Marshall, Rebecca Blaylock, Clare Murphy, Julia Sanders

**Affiliations:** 1 Centre for Reproductive Research and Communication, British Pregnancy Advisory Service (BPAS), London, EC4A 1JQ, UK; 2 School of Healthcare Sciences, Cardiff University, Cardiff, CF14 4XN, UK

**Keywords:** Risk, fertility, pregnancy, media, science communication, health

## Abstract

**Background: **The UK print and online media is an important channel by which scientific research is communicated to the public. Media risk messages relating to pregnancy or fertility contribute to the context of reproductive decision making, but their fidelity to the underlying science has been questioned.

**Method: **We measured the volume, distribution and content of science-based risk headlines relating to pregnancy or fertility in the UK media over four months. We grouped headlines into unique stories and categorised them by exposure and outcome of interest. We selected four unique stories for closer content analysis and assessed their fidelity to the underlying science, with attention to the role of press releases.

**Results: **We identified 171 headlines over four months (average 43 per month), comprising 56 unique stories. The unique stories most commonly concerned maternal risk factors (n=46) and child health outcomes (n=46). Maternal health outcomes were less frequently the focus (n=20). The most common risk factors in the media coverage were maternal food and drink (n=15), maternal medication and medical interventions (n=9), and maternal health factors (n=6). Media reports were largely faithful to press releases. Where substantive deviations from the underlying scientific study were identified, these could mostly be traced back to press releases or quotes from the study’s authors. Press releases often omitted caveats which were reinstated at the media reporting stage, alongside additional expert criticism.

**Conclusions: **Frequent science-based risk messages in the UK media frame mothers as vectors of potential harm to children, who are the focus of health outcomes. Largely, the media does not introduce misinformation, but reports press releases faithfully with additional caveats and expert commentary. Press releases fulfil an interpretative role, often omitting caveats and introducing new elements and advice to women. Their role as a bridge between scientific and lay audiences is discussed.

## Introduction

The UK media is an important channel through which new health research is disseminated to the general public ^1– 3^. Health stories are a particular staple of UK media reporting and are frequently communicated in the form of ‘risk messages’, wherein common exposures, habits, or lifestyle characteristics are positioned as risk factors for certain health outcomes ^2, 3^. Science-based health messages in the media influence the public’s understanding of health risks and their health-related decisions and behaviours ^4– 6^.

**Figure 1.**
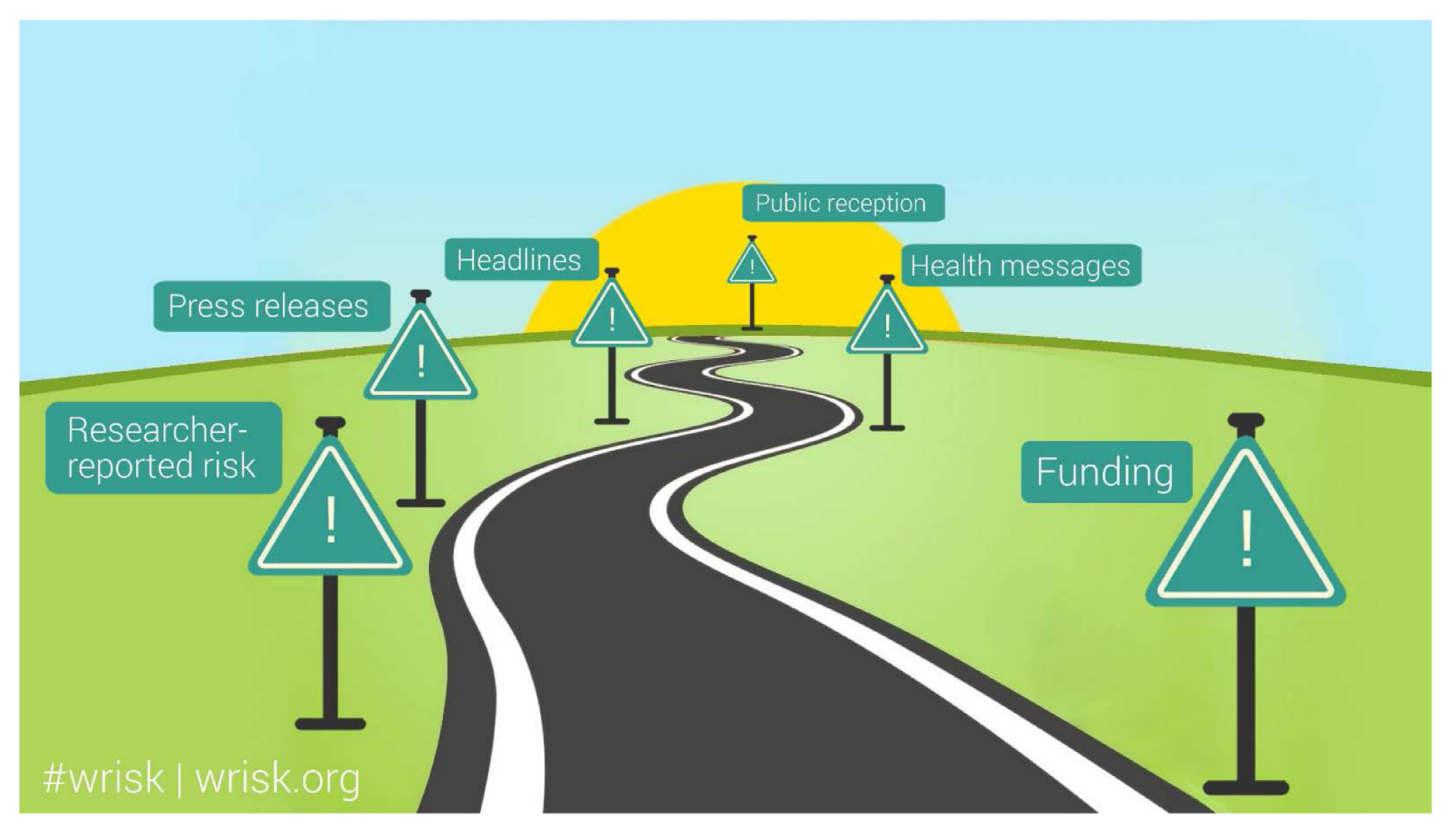
The risk reporting pathway (Wrisk project, 2020) ^21^.

Scientific studies are commonly communicated to journalists via press releases (PRs), which bridge the academic and media communities, enabling research institutions to generate coverage, and journalists to produce swift copy that conveys complex findings to a lay readership ^1^. PRs thus provide an important step on the risk reporting pathway between scientific papers and media reports ( Figure 1).

Concerns have been raised that the media’s reporting of health science is sensationalist, inaccurate, and undermines the intended messaging of the scientific community ^1, 3, 7, 8^. A number of studies have examined the role of PRs in science communication, and found they may be responsible, especially through the removal of caveats or the framing of correlational associations as causative ^1, 2, 7– 9^. While a certain degree of interpretation is necessary for PRs to fulfil their bridging function, such changes in the framing of risk messages at the PR stage are likely to later influence their public interpretation and reception, and therefore risk a detrimental impact on public health ^2, 4, 10, 11^.

Pregnancy risk is an area of contemporary debate and attention. Previous research has found that pregnancy is increasingly portrayed as a high-risk state, during which women must be closely surveilled, not for the sake of their own health but to protect the fetus from risks introduced by their behaviour ^12– 16^. This narrative can be traced back to the thalidomide disaster of the 1960s ^17^. Its reinvigoration in recent years has partly been fueled by research into the developmental origins of health and disease (DOHaD), which has recently focused on the impact of chemical exposures in utero on fetal health outcomes ^18– 20^.

This paper anlayses risk messages in the UK media relating to pregnancy and fertility, to better understand the context that informs women’s reproductive health decisions and behaviours, and to assess the extent to which (if at all) pregnant women are framed as vectors of potential harm (or benefit) to their fetus. Secondly, the fidelity of media reports to the underlying science is assessed through closer analysis of four illustrative examples. For each example, the communication of risk messages along the risk reporting pathway is examined, with particular attention to the role of PRs.

## Objectives

1. **Understand the landscape**: Describe the volume, distribution and content of science-based risk messages relating to pregnancy or fertility, as reported in UK-based print and online media outlets, over selected time periods.
2. **Assess reliability**: For selected news stories based on a scientific study, describe and map the risk reporting pathway(s) “from study to story”, by tracking key themes and informational elements across the original study manuscript, PR and media reports.

## Methods

### Objective 1: Understand the landscape

We used publicly available media reports. There were no particular ethical considerations.

**Selection of relevant time period(s)**

We randomly selected one month from each of the most recent four quarters (Q4, 2018 – Q3, 2019) for analysis, to accommodate possible seasonal variations in media reporting. The months selected were November 2018, and February, May and August 2019 (the second month of each quarter).

**Search of media database**

Using the LexisNexis news database, headlines published in UK print or online media outlets during the selected months were searched for relevant key words ( *"babies" or "unborn" or "pregnant" or "pregnancy" or "fertility" or "miscarriage" or "stillbirth" or "stillborn").*

**Exclusion process**

**Figure 2.**
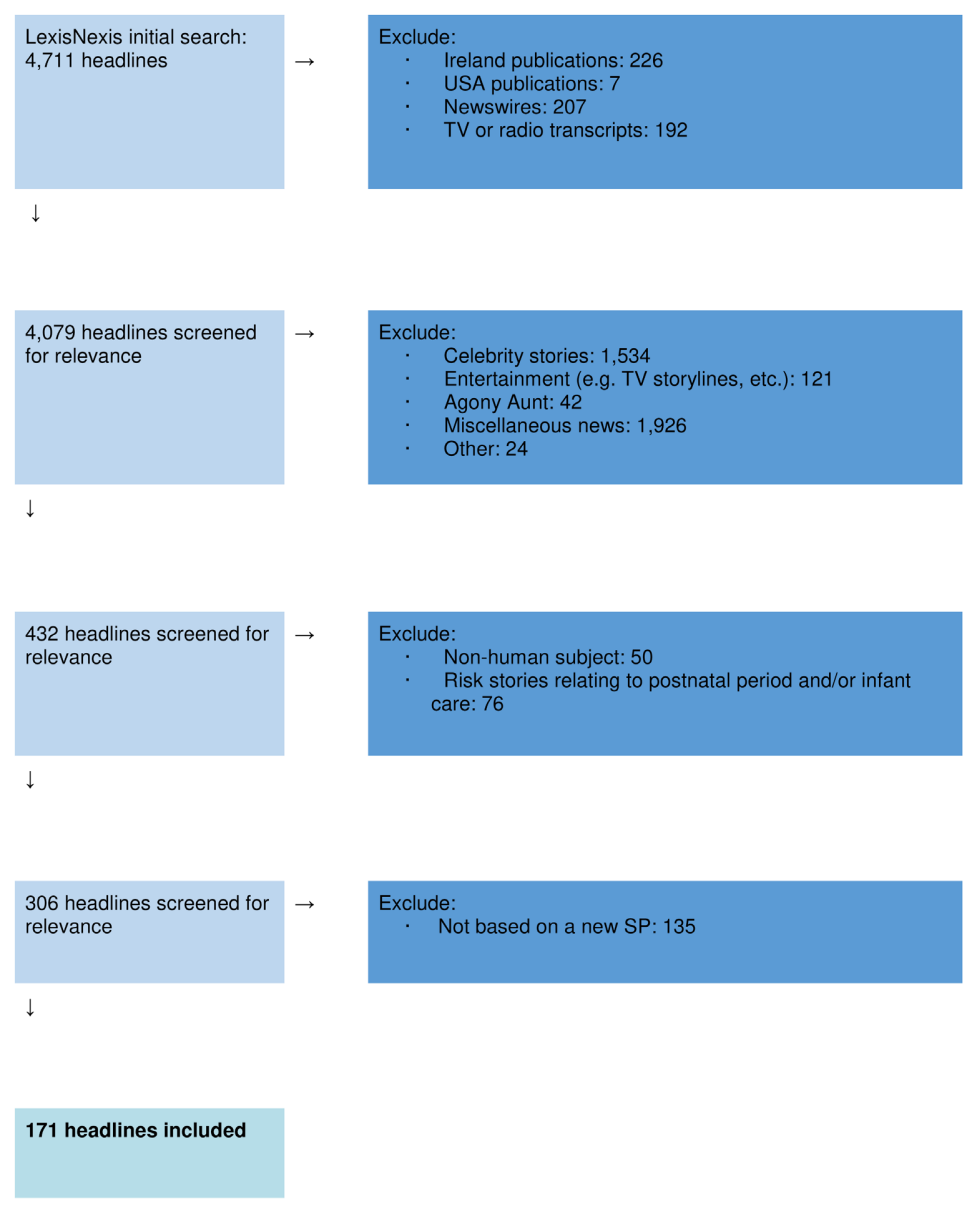
Exclusion flow diagram.

The inclusion and exclusion criteria were agreed by two researchers (OM and RB). The full exclusion process is summarised in Figure 2.

**Inclusion criteria**: Headlines relating to a social, environmental, or clinical risk to fertility, a pregnant woman or her offspring, and based on a new scientific study (including research conducted/published outside of the UK).

**Exclusion criteria**: Headlines from newswires, broadcast media or media outlets with a predominant readership outside of the UK; headlines with no risk element (including celebrity, entertainment, or miscellaneous news stories); headlines about the postnatal period or infant care; and headlines not based on a new scientific study.

**Analysis**

Stories were deemed to be based on a scientific paper if they mentioned a new scientific study or research, and the corresponding paper was identified and noted based on its key findings, authors and date of publication. We grouped media reports based on the same scientific study into “unique stories” and assigned a Unique Story ID (USID). For each month we calculated the number of headlines and unique stories. We calculated the number of headlines per unique story, and the mean and median overall.

We coded each unique story according to the exposure and outcome of interest. We categorised exposures by risk locus (maternal; paternal; offspring; other) and by topic. We categorised outcomes by outcome locus (maternal; paternal; offspring; other). Two researchers (OM and RB) undertook this process independently and established and a consensus. Some unique stories were assigned to multiple categories as appropriate. For each category we calculated the number of relevant unique stories. We used Microsoft Excel (v2103) for our analysis.

### Objective 2: Assess reliability

Selection of illustrative examples

We selected the unique story that generated the most headlines in each month and assessed the reliability of associated media report. Four unique stories selected were based on the following studies:

November 2018: McQuire, C, Mukherjee, R, *et al.* (2019). Screening prevalence of fetal alcohol spectrum disorders in a region of the United Kingdom: A population-based birth-cohort study. *Preventive Medicine*, 118, 344–351. doi: 10.1016/j.ypmed.2018.10.013

February 2019: Golding, J, Gregory, S, *et al.* (2019). Maternal prenatal external locus of control and reduced mathematical and science abilities in their offspring: A longitudinal birth cohort study. *Frontiers in Psychology*, 10(Feb) doi: 10.3389/fpsyg.2019.00194

May 2019: Gignac, F, Romaguera, D, *et al.* (2019). Maternal nut intake in pregnancy and child neuropsychological development up to 8 years old: a population-based cohort study in Spain. *European Journal of Epidemiology*, 34(7), 661–673. doi: 10.1007/s10654-019-00521-6

August 2019: Zhang, T, Sidorchuk, A, *et al.* (2019). Association of Cesarean Delivery With Risk of Neurodevelopmental and Psychiatric Disorders in the Offspring: A Systematic Review and Meta-analysis. *JAMA network open*, 2(8), e1910236. doi: 10.1001/jamanetworkopen.2019.10236

Collating of materials

We collated the following documents for each illustrative example:

- Original study manuscript
- PR as published by the university or research institution (where applicable)
- Communications about the study published by the Science Media Centre (SMC) (where applicable)
- Print and online media coverage across UK media outlets

To fulfil the objective of assessing media accuracy, we considered it appropriate to analyse all the media coverage of these four unique stories, including any articles not captured by our original search. This was especially important since previous research has indicated that as an individual risk story ages, the tone of the media discourse may evolve ^2^. To ensure completeness, we conducted additional searches using the LexisNexis database, with wider date parameters and more specific keywords (for example “FASD”, etc.). Once we had collated all media reports, we removed duplicates (defined as an identical article published by the same news outlet on the same day).

Coding of media reports, PRs and studies

Following methodological precedent from Lee, Sutton and Hartley (2016) and Reisch and Spiegelhalter (2011), for each illustrative example we identified and coded key themes and informational elements in the original study, PRs, and media reports. Two researchers (OM and RB) extracted this information independently and agreed a summary by a process of consensus.

The information extracted was grouped under four broad headings:

1. Description of study and findings (including study design, exposure and outcome of interest, use of statistics, description of association, use of language, etc.)

2. Caveats, criticism or study limitations (e.g. a specific statement that causation was not established)

3. Associated discussion topics (framing)

4. Advice, warnings, or reassurance to the public (both direct and indirect)

Mapping and descriptive analysis

For every document, we noted the presence or absence of each theme or informational element, and calculated the number of media reports containing each theme or informational element. This allowed us to compare the content of the original study, PR and media reporting.

We undertook a descriptive analysis to trace the themes and informational elements along the risk reporting pathway, according to the four groupings outlined above.

## Results

### Objective 1: Understand the landscape

Volume and distribution of headlines and unique stories

**Figure 3.**
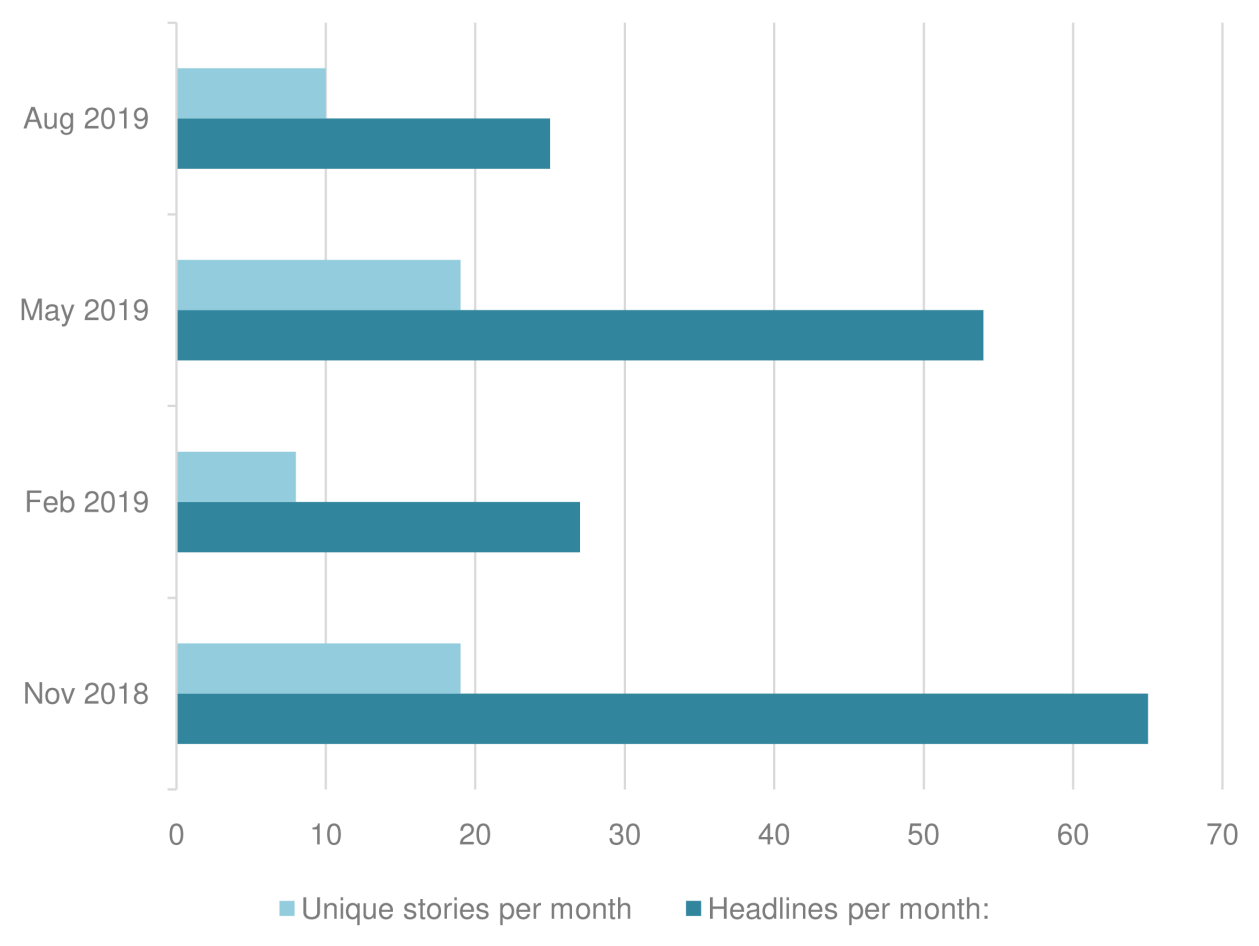
Unique stories and headlines per month.

171 headlines were identified that met the inclusion criteria, which were mostly concentrated in November 2018 and May 2019. Analysis of the 171 articles found that they comprised 56 unique stories (based on 56 research studies). Again, the unique stories were concentrated in November 2018 and May 2019 ( Figure 3). Per month, the mean number of headlines was 43 and unique stories 14.

**Figure 4.**
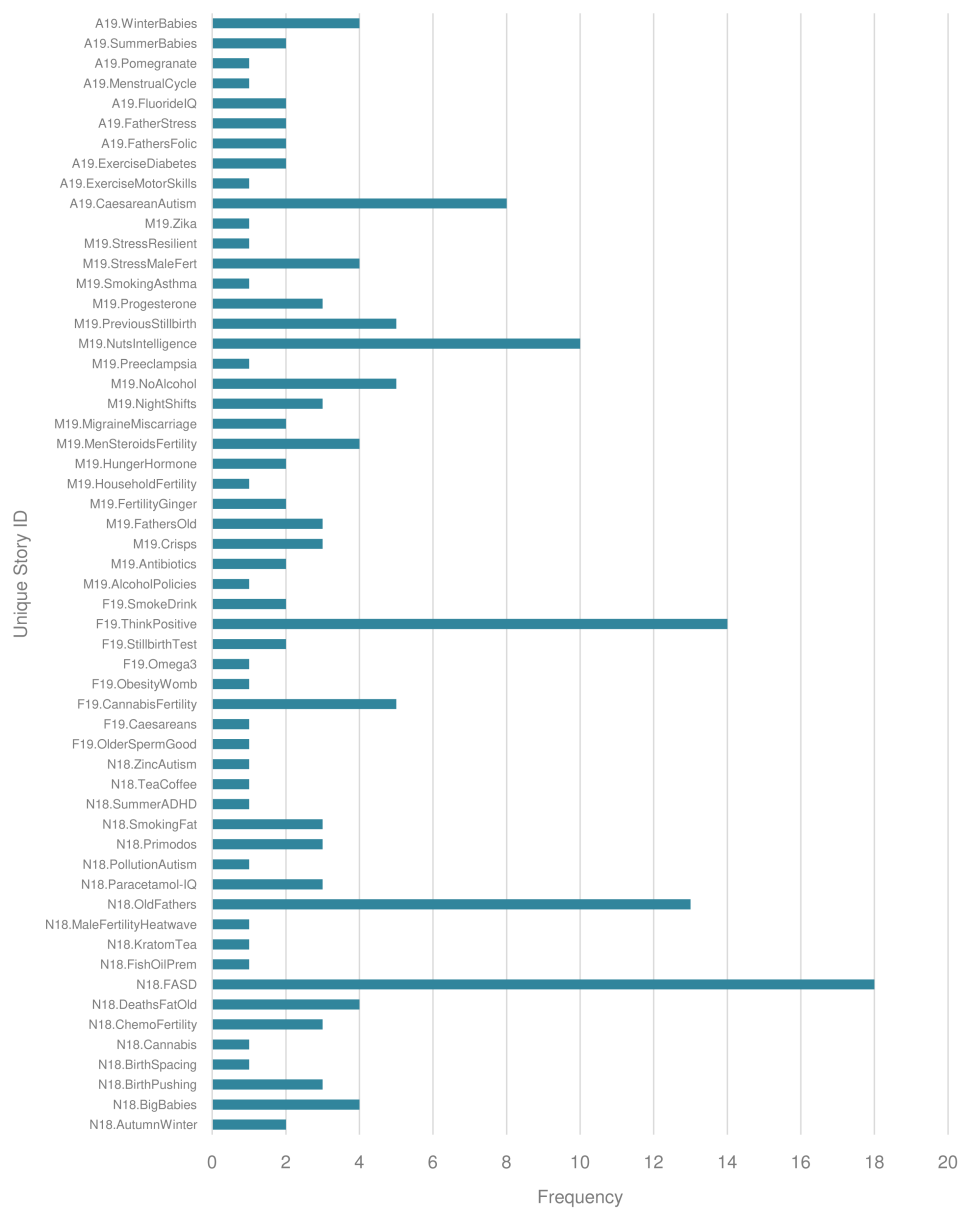
Headlines per unique story.

Unique stories generated varying numbers of headlines. The median number of headlines per unique story was 2, but some stories achieved significantly more headlines, as illustrated in Figure 4.

Content of unique stories

**Figure 5.**
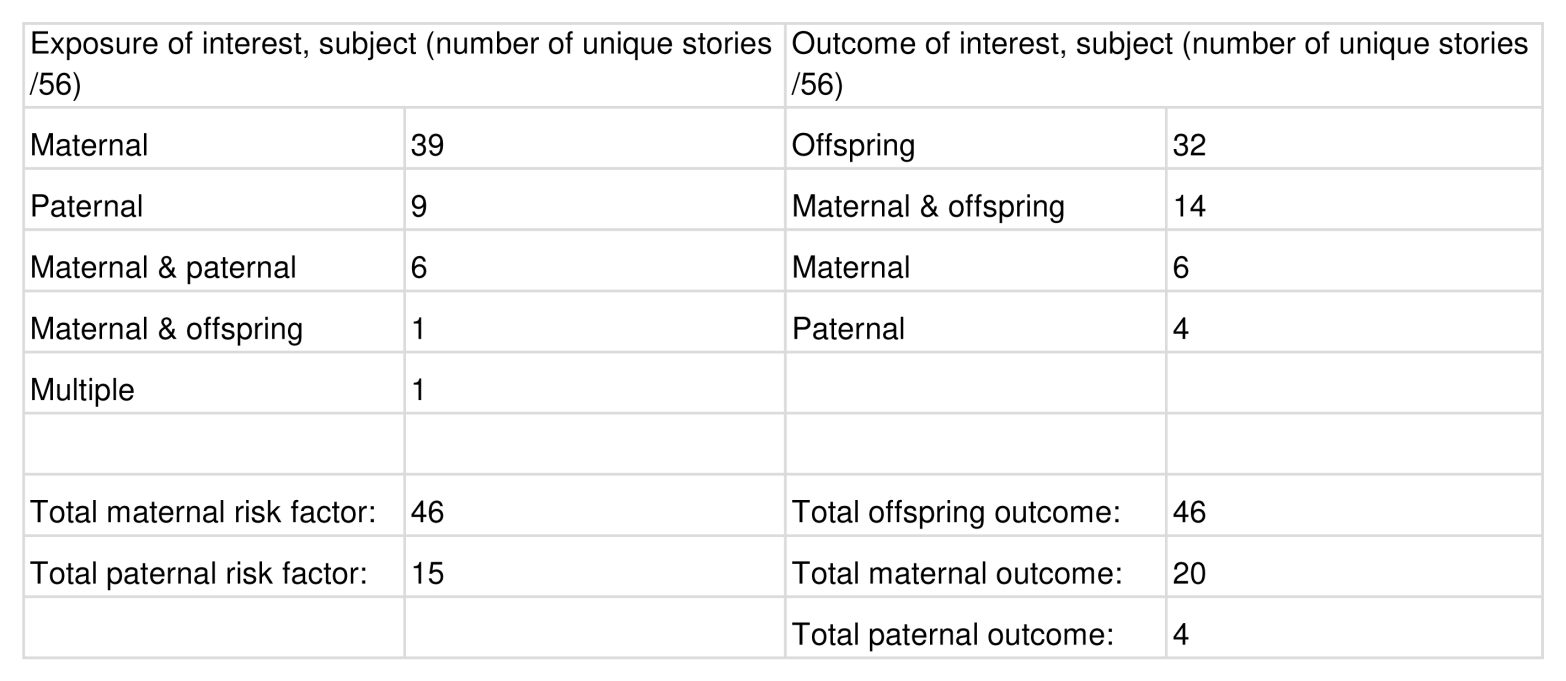
Exposure and outcome of interest, categorised by subject.

Of the 56 unique stories, maternal risk factors (n=46) and child health outcomes (n=46) were most commonly identified. Maternal health outcomes were less frequently the focus (n=20). The categorisation of exposures and outcomes by subject is given in Figure 5.

**Figure 6.**
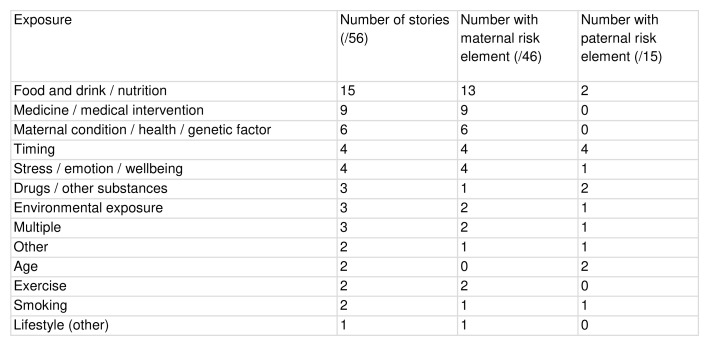
Exposure of interest, categorised by topic.

The most common risk factors were maternal food and drink (n=13), maternal medication and medical interventions (n=9), and maternal health factors, including genetic factors or underlying conditions (n=6). The categorisation of exposures by topic is given in Figure 6.

### Objective 2: Assess reliability

**Illustrative example 1**

Documents:

- Study: McQuire, C., *et al.* (2019)
- Press release: University of Bristol (2019). *First UK prevalence estimate FASD*
- Science Media Centre (2018). *expert reaction to screening study on uk prevalence of fetal alcohol spectrum disorders (FASD)*
- Science Media Centre (2018). *uk prevalence of fetal alcohol spectrum disorders*
- Media reports (n=16)

Study summary:

Novel screening algorithms were applied to data from the ALSPAC longitudinal birth cohort study to estimate the screening prevalence of fetal alcohol spectrum disorder (FASD). Different missing data strategies were evaluated to yield prevalence estimates ranging from 6–17%.

**Figure 7.**
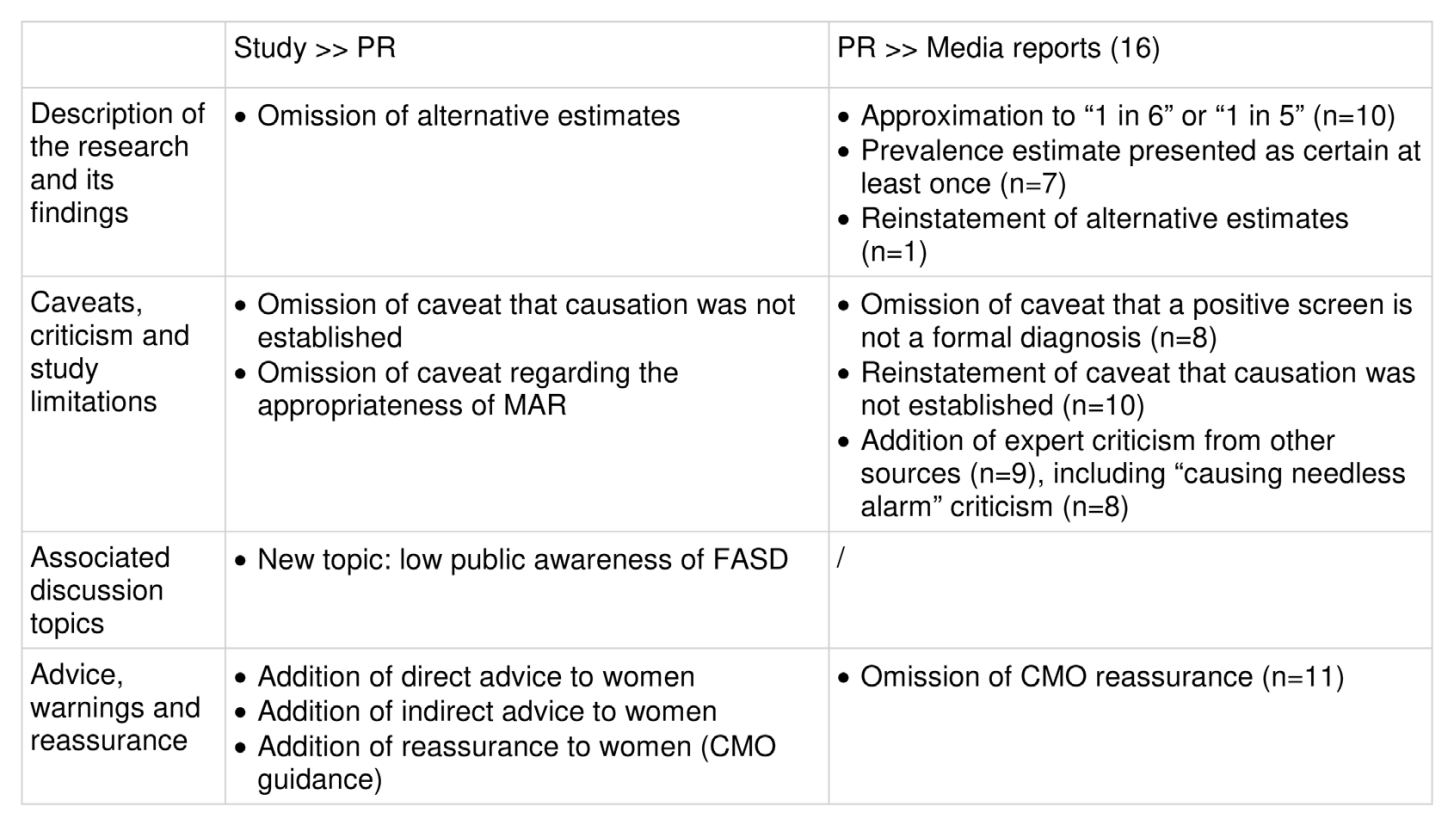
Key changes along the risk reporting pathway (illustrative example 1).

Comparison of study, PR and media reports:

Key changes at each stage of the risk reporting pathway are summarised in Figure 7.

**Description of the research and its findings**: The study’s design, exposure and outcome of interest were accurately described in the PR and in 15 of the 16 media reports. The one remaining report included a passing reference to one of the study’s secondary findings. The highest prevalence estimate of 17% was quoted accurately in the PR and the majority of media reports (n=13), but eight reports also approximated this to “one in six”, and two to “one in five”, in their headlines. The PR used appropriate language to convey uncertainty (e.g. “features consistent with FASD”; “children could have symptoms”; “up to 17%”) and presented the figure of 17% as an estimate throughout, including in the headline. Three quarters of media reports (n=12) made clear somewhere in the report that the figure was an estimate, but seven also presented the figure as certain at least once, in statements such as "17% of babies are harmed by mothers' drinking". The PR did not state that the prevalence estimate had varied by screening method or include the lower estimates (which had been obtained via different imputation methods for missing data). Only one media report (in The Guardian) reinstated this information.

**Caveats, criticism, and study limitations**: The caveat that a positive screen for FASD is not the same as a formal diagnosis was present in the study, the PR and half of the reports (n=8). The caveat that a causative association had not been established (between prenatal alcohol exposure and the developmental outcomes recorded) was omitted in the PR, but reinstated in the majority of media reports (n=10). Ten media reports included additional expert criticism sourced from either the Science Media Centre (n=5) or a press comment issued by the British Pregnancy Advisory Service (BPAS) (n=10), including a criticism that the study was “causing needless alarm”, which was cited in eight reports. Overall, the majority of media reports (n=10) included at least one caveat or critical statement, with most including three or more (n=9). Of the six media reports that included no criticism or caveats, two were very short (50 words or less) and two were passing references to the study in articles published several months later.

**Associated discussion topics**: The PR developed discussion topics from the study, including UK rates of prenatal alcohol exposure (PAE); the prevalence of binge drinking among pregnant women; conceptualising FASD as an under-diagnosed or “hidden” condition; and framing FASD as a significant public health concern. The PR also introduced one new topic, low public awareness of FASD, which was mentioned in half of the media reports (n=8). The discussion topics in the media coverage closely mirrored those in the PR, especially the placement of FASD as a public health concern and discussion of PAE rates in the UK. 

**Advice, warnings and reassurance**: The PR introduced direct advice to women in a quote by the study’s lead author, who referred to guidance that the safest approach is to abstain from alcohol. Two paragraphs at the end of the PR also summarised advice from the UK’s Chief Medical Officer (CMO), which affirms this but also offers reassurance by stating that those who have drunk before realising they are pregnant are unlikely to have caused harm. The majority of media reports (n=11) included direct or indirect advice to women, but few included the CMO’s reassurance (n=5).

**Summary**: The PR described the study accurately with appropriate language to convey uncertainty, but failed to include alternative estimates for FASD prevalence and some key caveats. The PR introduced both advice and reassurance to women through quotes from the main author and others, including the CMO. The media coverage also communicated the study's findings effectively, but often approximated the prevalence estimate of 17% to “one in six” or “one in five”, and occasionally presented it as certain, implying a level of confidence that the study authors had not claimed. The majority of media reports addressed the study’s limitations through the inclusion of caveats and criticisms, including caveats that had been omitted in the PR, and additional criticism sourced from the SMC and BPAS.

**Illustrative example 2**

Documents:

- Study: Golding, J., *et al.* (2019)
- Press release: University of Bristol (2019). *Thinking positive during pregnancy?*
- Media reports (n=16)

Study summary:

Data from the ALSPAC longitudinal birth cohort study were analysed to assess the association between maternal locus of control (LOC), measured during pregnancy, and children’s reasoning skills in maths and science. Maternal external LOC was associated with poorer academic performance in children. Three separate sets of factors were controlled for, to assess the extent to which they mediated the association. Taken together they identified at least 50% of the mechanism.

**Figure 8.**
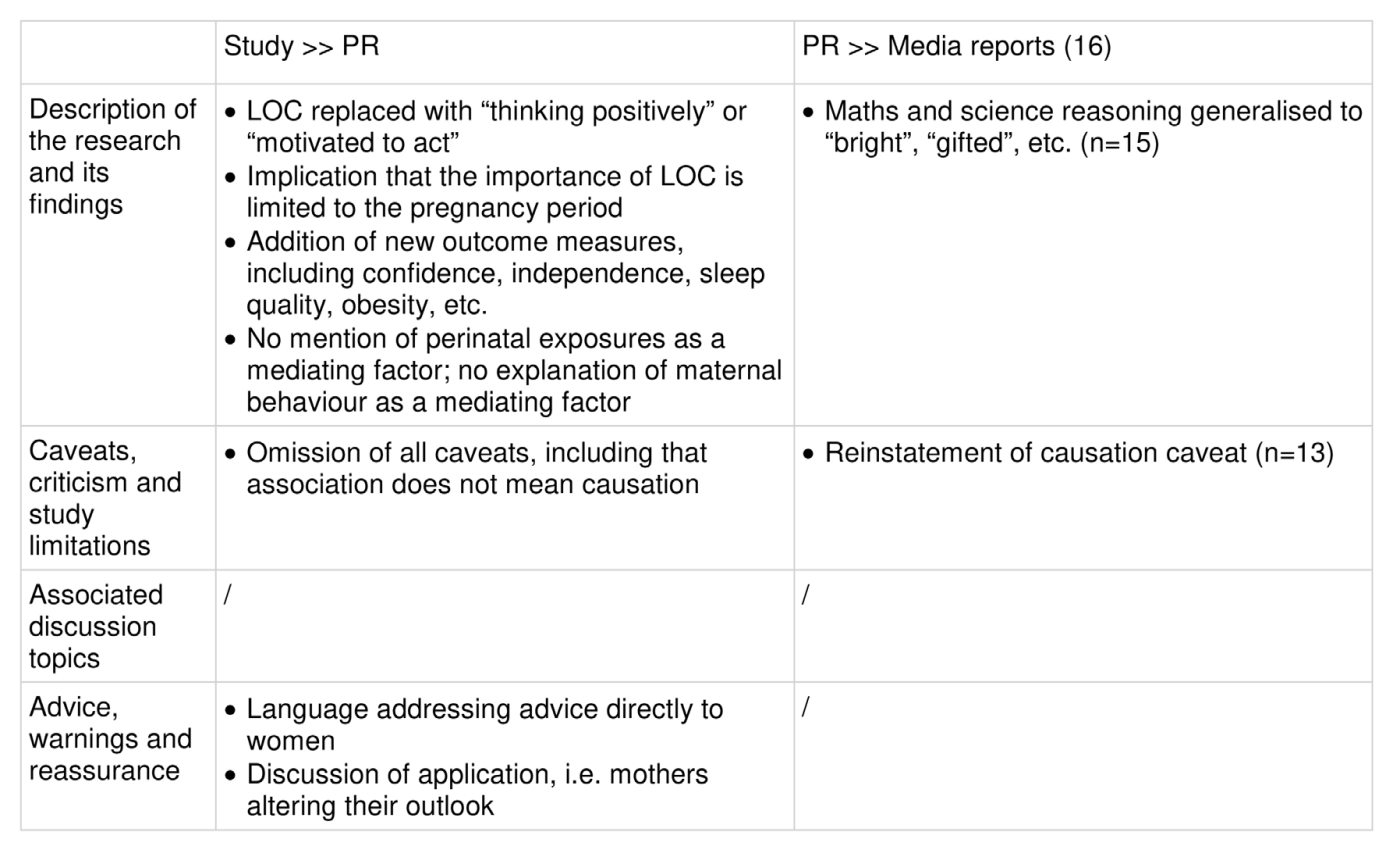
Key changes along the risk reporting pathway (illustrative example 2).

Comparison of study, PR and media reports:

Key changes at each stage of the risk reporting pathway are summarised in Figure 8.

**Description of the research and its findings**: The study design was reported accurately in the PR and all 16 media reports. However, there were linguistic differences in the descriptions of the exposure and outcome of interest. The exposure discussed in the study was maternal LOC, which is defined as a personality scale that “identifies individuals’ general attitude to what happens to them as largely a matter of luck or fate or of powerful others (externality) or whether they feel they can influence the consequences (internality)”. However, the press release presented this as “thinking positively during pregnancy”, which is arguably inaccurate, since it implies optimism rather than a sense of control. All 16 media reports mentioned both LOC and “thinking positively”, but the latter was more prominent, appearing in almost all the headlines (n=14). The PR also stated that people with an external LOC are less “motivated into action” or believe there is “little point in making an effort”, which was echoed in 14 media reports. The temporal nature of LOC is also presented misleadingly in the PR. In the study, LOC is understood as a consistent personality characteristic, which was measured during pregnancy as a baseline. Indeed, its manifestation in parenting behaviours long after pregnancy is a primary avenue of enquiry for the authors. Nonetheless, the PR fails to explain this, and instead only discusses maternal attitude *during* pregnancy, implying that the importance of LOC is limited to those nine months, and that a mother’s mindset during pregnancy can impact her children for years to come regardless of her future behaviours.

The outcome of interest, maths and science reasoning, was reported accurately in the PR and all 16 media reports, although almost all reports used more generalised language such as “bright”, “gifted” or “the next Einstein”. However, the PR introduced additional outcomes which had not been included in the study, including children’s health, confidence, independence, sleep, emotional control, diet, school-related social difficulties, and obesity. All of these additional outcomes were introduced via quotes from two of the study’s authors, despite bearing no clear relevance to the findings of this particular study. These outcomes were mentioned in almost all (n=14) media reports.

A primary objective of the study was to assess the extent to which the association is mediated by factors including perinatal exposures and parenting behaviours, which were found to explain more than half of the mechanism. Some of these parenting behaviours were mentioned in the PR, but their role as mediators of the association was not discussed. This was also absent from all 16 media reports.

**Caveats, criticism and study limitations**: The PR does not mention any of the study’s limitations or caveats, including the differentiation of association and causation. However, the majority of media reports (n=13) did reinstate this caveat.

**Associated discussion topics:** The primary discussion topic in the media reports was past research on LOC. This theme was present in both the PR and the study itself.

**Advice, warnings and reassurance**: The PR introduced direct advice to the general public. Even the phrasing of its headline and opening sentence (“Thinking positively during pregnancy?”; “Your attitude during pregnancy…” etc.) is clearly intended to address women directly. There is also discussion in the form of quotes from the study’s authors about mothers changing their outlook in order to improve their children’s outcomes. Almost all (n=14) media reports included indirect advice through discussion of mothers altering their LOC, mainly through the inclusion of these quotes from the PR.

**Summary:** The press release altered the description of the exposure of interest to “thinking positively during pregnancy”, arguably misrepresenting the meaning of LOC and implying that its importance is limited to the nine months of pregnancy. Several child outcomes that were not explored in the study were added to the PR via the inclusion of author quotes. Additionally, the PR did not discuss the study’s limitations or state that causation had not been established, and there was no discussion in the PR of parenting as a mediating factor of the association. The press coverage echoed the PR closely with two exceptions: the generalisation of the outcome of interest (maths and science reasoning) to “bright”, “gifted” etc., and the reinstatement of the caveat that causation was not established in almost all reports (n=13).

**Illustrative example 3**

Documents:

- Study: Gignac, F., *et al.* (2019)
- Press release: IS Global (2019). *Maternal Nut Consumption During Pregnancy Linked to Improvements in Neurodevelopment in Children*
- Media reports (n=17)

Study summary:

Data from the Spanish Childhood and Environment (Infancia y Medio Ambiente, INMA) Project, a population-based birth cohort study, were analysed to assess whether maternal nut consumption during pregnancy is associated with child neuropsychological outcomes. First-trimester nut consumption was associated with improved child performance in four cognitive tests. After adjusting for confounders, reduced hit reaction time standard error in the Attention Network Test (ANT), a measure of sustained attention, remained significant. Third trimester nut intake showed a weaker association.

**Figure 9.**
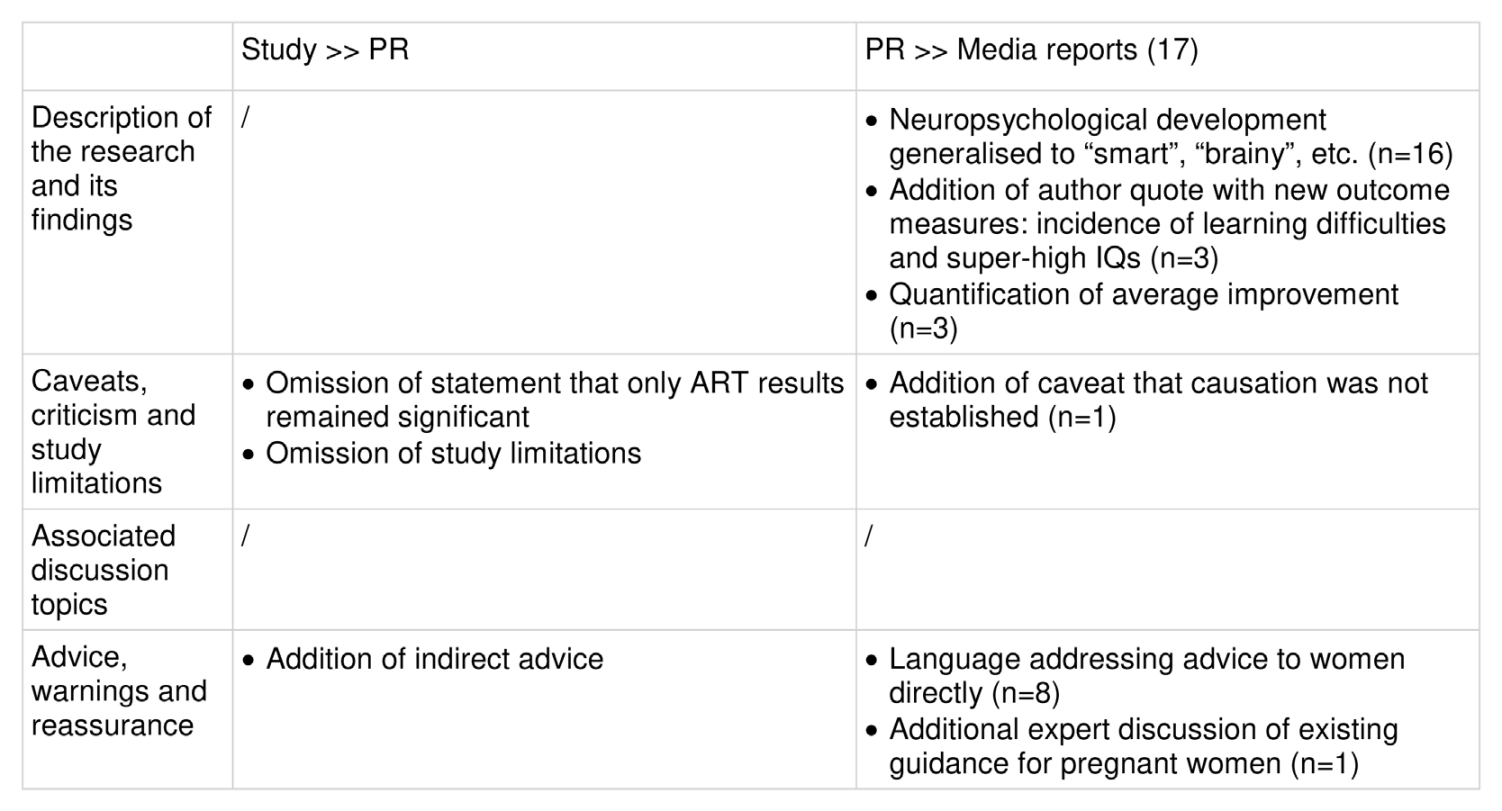
Key changes along the risk reporting pathway (illustrative example 3).

Comparison of study, PR and media reports:

Key changes at each stage of the risk reporting pathway are summarised in Figure 9.

**Description of the research and its findings**: The study’s design and the exposure of interest (maternal nut intake) were described accurately in the PR and media reports. All 17 media reports mentioned maternal nut intake, with the majority specifying the first trimester of pregnancy and giving details of serving size and/or frequency of consumption (n=13 and n=10 respectively).

For the outcome, similarly to the previous examples, almost all the media reports (n=16) used simpler language, expressing neuropsychological development as “intelligent”, “smart” or “brainy”, although most (n=12) also mentioned the specific facets measured, i.e. attention, memory, or cognitive function. Three media reports introduced additional outcomes, the incidence of learning difficulties and super-high IQs, which had not been mentioned in either the study or the PR. These were included in the form of a quote from one of the study’s authors, which was not present in the PR. The same three media reports also stated that child performance in cognitive tests had improved by an average of 3%, a figure that is not given in either the PR or the study.

**Caveats, criticism and study limitations**: The study was clear that while improvements were observed in all cognitive tests, only one (hit reaction time standard error in the ART) remained significant after fully adjusting for confounders. However, this is not specified in the PR, which describes in its by-line “better outcomes after birth in cognitive function, attention capacity and long-term working memory”. None of the media reports explained that only the ART results were significant after adjusting.

The PR omitted any discussion of the study’s limitations, but it did state that the findings should be interpreted with caution. Only three media reports included any caveats in their reporting of the study. One media report included the caveat that causation had not been established since this was an observational study, which was not stated explicitly in either the study or PR (although both acknowledged that further research is required to confirm the findings).

**Associated discussion topics:** Four media reports discussed maternal nutrition and the benefits of eating nuts more generally. Both these topics were present in the study and PR. 

**Advice, warnings, and reassurance**: Just under half of the media reports (n=8) used language that addressed advice to women directly, for example, “eat nuts for a brainy baby” or “mothers told” to eat nuts. This direct language was not present in the study or PR, but the PR did contain indirect advice through discussion of the benefits and the ideal amount of nuts to eat. One media report sourced an expert quote from the Royal College of Obstetricians and Gynaecologists, which discussed the existing guidance on nut consumption during pregnancy.

**Summary:** The PR generally reflected the study’s findings accurately, but omitted caveats, which remained absent in the majority of media reports. Media reports sometimes introduced direct advice, and almost always generalised the outcome of interest to “brainy”, “smart”, etc., but otherwise the majority reported the study’s findings accurately. However, three media reports introduced a new figure (3% average improvement) alongside two new outcome measures (incidence of learning difficulties and super-high IQs) which had not been present in the study or PR. All three appeared to source the latter information from one of the study’s authors. It is unclear whether the 3% figure was obtained from the same source.

**Illustrative example 4**

Documents:

- Study: Zhang, T., *et al.* (2019)
- Science Media Centre (2018). *expert reaction to study on caesareans and neurodevelopmental disorders*
- Media reports (n=16)

*NB no PR was published to accompany this study.*

Study summary:

A systematic review and meta-analysis of 61 studies, comprising over 20 million births, found that caesarean birth was significantly associated with both autistic spectrum disorder (ASD) and attention-deficit/hyperactivity disorder (ADHD) in children.

**Figure 10.**
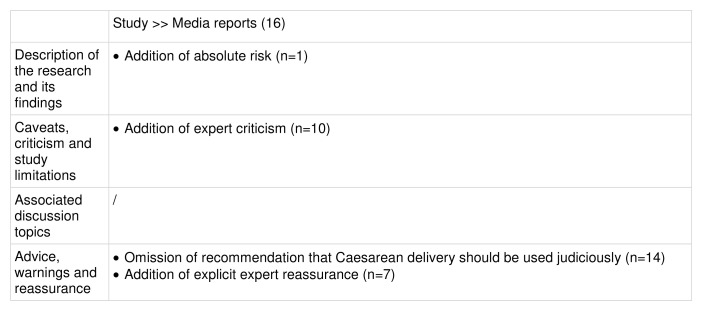
Key changes along the risk reporting pathway (illustrative example 4).

Comparison of study and media reports:

Key differences between the study and media reports are summarised in Figure 10.

**Description of the research and its findings**: All 16 media reports described the study accurately, reporting the exposure and outcome of interest correctly. The majority of reports specified the relative increased risk for ASD and ADHD (n=14 and n=13 respectively). One media report also specified the increase in absolute risk for ASD, which was not mentioned in the study but discussed in the SMC expert reaction summary.

**Caveats, criticism and study limitations**:

The majority of media reports (n=10) conveyed caveats given in the study. Of the six that did not, five were passing references to the study in articles about caesarean births published several weeks after the study’s publication. Ten reports stated explicitly that causation had not been established, with one including this caveat in the headline. Half of the reports (n=8) included the caveat that the mechanism by which caesarean birth is associated with ASD and ADHD is not understood, and eight mentioned confounding factors that could explain the link. Five reports stated that Caesarean delivery is sometimes necessary to reduce other risks. Most reports (n=10) also added expert criticism sourced from outside the PR (mostly from the SMC summary).

**Associated discussion topics:**

Half of the media reports (n=8) discussed the rate of caesarean births and seven discussed previous research on caesarean births. Both topics of discussion were present in the study.

**Advice, warnings and reassurance**:

No media reports advised that caesarean births should be avoided, and most (n=14) omitted the recommendation from the study that caesarean delivery should be used judiciously. Nearly half of the media reports (n=7) included explicit reassurance from experts, sourced from the SMC, that women should not be alarmed by the findings, with one report including this in its headline.

**Summary:** All media reports described the findings accurately, with the majority specifying the relative increase in risk for both ASD and ADHD. Ten reports included caveats from the study and also included expert commentary from other sources. No warnings or advice were added by the media, and in fact the study’s recommendation that caesarean delivery should be used judiciously was mostly omitted. Instead, expert reassurance to women was included in seven reports, including once in the headline.

## Discussion

We found that risk messages relating to pregnancy and fertility are a common feature of UK print and online media reporting, appearing in 43 headlines per month on average – more than once per day. Each month there were, on average, 14 unique stories based on a new scientific study, suggesting that roughly every other day a novel scientific finding regarding pregnancy or fertility risk is presented to the public. These messages contribute to the context of pregnant women’s health-related decisions. Since the majority of the science-based unique stories we identified concerned lifestyle or health factors, particularly food and drink, medication or underlying health conditions, their cumulative effect may increase anxiety among the pregnant population that any habit, lifestyle factor, or even emotion they experience during pregnancy is now considered a risk factor.

The majority of the unique stories focused on risk factors relating to the pregnant woman, which were framed in most cases only in terms of their possible impact on fetal health, without discussion of the health outcomes (either positive or negative) for the woman. The separation of maternal and child health outcomes in this way positions pregnant women as vectors of potential harm or benefit to their fetus, and frames their health status, behaviours and mindset during pregnancy as determinants of offspring wellbeing, both immediately and long into the future. Previous authors have raised concerns about this positioning of women as vectors, which has been used to justify increasing surveillance and scrutiny of pregnant women’s choices without due consideration of their autonomy ^12– 16^. 

PRs had a strong influence on media coverage, indicating that academic institutions have a large degree of control over the media’s reporting of their research. Our findings support previous research that exaggeration in PRs is echoed in media reports ^1, 8^. To a degree, journalists fulfilled a corrective role, reinstating caveats omitted at the PR stage and sourcing additional expert criticism. However, certain misleading elements introduced at the PR stage (such as the framing of external LOC in example 2 as “thinking positively during pregnancy”) remained present in the media coverage, and some caveats or other pertinent contextualising information (such as the lower FASD estimates in example 1, or the role of parenting behaviours in example 2) were not recovered at the media stage to the risk reporting pathway. Since media reports inform the public’s health behaviours, understanding and decisions ^4– 6^, these inaccuracies matter. The portrayal of external LOC as “thinking positively during pregnancy”, for example, may conceivably cause women who have difficult pregnancies or antenatal depression to feel that they have caused irreversible harm to their child, and this is compounded in that example by the lack of explanation in the PR and media reports that parental behaviour is a mediating factor of the association. Efforts at the PR stage to ensure that accuracy, caveats and context are preserved may help to avoid such instances of needless anxiety.

PRs were not the only route by which information reached journalists: several media reports included additional expert commentary sourced from the SMC, other experts, or directly from the study authors. In illustrative example 4, which had no PR, we observed a noteworthy reliance on expert quotes sourced from the SMC and elsewhere, suggesting that in the absence of a PR journalists may choose not to add interpretation themselves, preferring to rely on expert opinion. Conversely, in illustrative example 1, the SMC materials – which were highly critical of the study – were not frequently used. This may indicate that time-pressed journalists are less compelled to consult additional sources for interpretative expert commentary if a comprehensive PR is available. If true, this would constitute another way in which PRs exert influence on media reporting. Reduced journalist reliance on external expert commentary is material because, in our examples, the heavy use of external sources appeared to yield more accurate and reassuring reporting. The media coverage in illustrative example 4 deviated the least from the underlying study, and was particularly measured and reassuring, incorporating expert quotes that emphasised the study's limitations and explicitly told women not to worry. Notably, the lack of PR in illustrative example 4 did not appear to hamper media coverage, and neither was coverage hampered by the publication of critical SMC round-ups: examples 1 and 4 each generated 16 headlines, despite the cautionary nature of the accompanying SMC commentaries.

To conclude, the PRs analysed here omitted caveats and other pertinent information, introduced advice to women, and occasionally included misleading elements. Greater reliance on SMC (and other third-party) expert materials appears conducive to reliable, measured reporting. We suggest that a new model of science dissemination may be desirable, whereby journalists are encouraged to consult expert opinion from a variety of sources or make greater use of comment round-ups compiled by independent third parties such as the SMC.

### Strengths and limitations

Our study did not analyse broadcast media or social media, both of which are important streams by which health information is disseminated. Our four illustrative examples were selected based on volume of coverage, which ensured a richness of material to analyse, but also rendered them “outliers” among our unique stories (which mostly only generated a small number of headlines) and therefore possibly atypical. Lastly, this is an observational analysis based on correlational relationships and an assumption that the risk reporting pathway was followed. It is possible that similarities between PRs and media reports were not causal and occurred by coincidence.

### Future research

The media landscape does not necessarily reflect the nature of research undertaken in the field, since not all studies are reported. Future research could analyse risk messages disseminated by journals or research institutions to examine whether the fetus-centric framing observed in the media is also present earlier on the risk reporting pathway. It would also be interesting to repeat this analysis for risk headlines relating to infant care, including breastfeeding. On the role of PRs, further research could compare media coverage of scientific papers published with or without a PR, to assess whether PRs alter the volume or nature of coverage. The use of SMC commentary by journalists could also be examined to see whether its uptake is reduced by the availability of a comprehensive PR, and whether its use generally leads to more balanced and/or reassuring reporting.

## Data availability

Figshare. Risk messages relating to pregnancy and fertility: a media content analysis (dataset). https://doi.org/10.6084/m9.figshare.14480784.v1 ^22^

Data are available under the terms of the Creative Commons Zero "No rights reserved" data waiver (CC0 1.0 Public domain dedication).
