# Supplementary material for: Risk messages relating to fertility and pregnancy: a media content analysis
Source: Wellcome Open Res. 2021 May 14;6:114. [Version 1] doi: 10.12688/wellcomeopenres.16744.1 (PMC8276184; doi:10.12688/wellcomeopenres.16744.1)
Supplement: Peer review history

# Peer review: Reviewer response for version 1

**DOI:** 10.21956/wellcomeopenres.18465.r43903

**Published:** 2021-07-12

**Authors:** Sarah Richardson

**Affiliations:** 1 Department of the History of Science and Studies of Women, Gender, and Sexuality, Harvard University, Cambridge, MA, USA

This qualitative study of media coverage of scientific claims about risks during pregnancy offers valuable insights into the uptake and circulation of such findings.  This is important because media uptake and dissemination of risk claims is critical to the processes by which pregnant people themselves decide whether to alter their behavior, public health policies are funded and crafted, and resources for research are directed.  Examining in-depth four examples from their sample of 171 headlines, the authors include a detailed analysis of the contributing role of institutional press releases featuring quotations from the original study authors to scientific media reporting that overstates the original findings.  This is an important finding because researchers often claim that they cannot control hyped media coverage of their studies and that any infidelities in reporting are a result of reporting errors on the part of media.

**Is the work clearly and accurately presented and does it cite the current literature?**

 The presentation is clear. The authors offer multiple accessible tables making the data and reasoning that they used transparent.  The paper is appropriately situated in the current literature. 

 Currently, the paper is written as a series of highly modularized case studies, so that the main body of the paper reads almost as if it is an outline.  The paper could be improved in clarity by narrating these case studies in prose and knitting them together in a more comparative format.

 Small comments:

- Figure 7: What is MAR?
- Several typos: "established and a consensus" and "agreed a summary" (pg. 5), study titles on pg. 6-7 and pg. 12 need an edit,  in Fig. 9 should ANT be ART?
- Note that the term "vectors" (pg. 13) to describe the positioning of women in maternal-fetal effects literature comes from Richardson 2015.

**Is the study design appropriate and does the work have academic merit?**

 Yes.  I have several small comments, however, that might strengthen the paper:

- The authors' second objective is described as to "Assess reliability."  This would be better rephrased as "Assess fidelity to the original scientific article," as the authors are not actually assessing how much you can rely on the science or the reporting of the science. 
- Figure 1 should be unpacked further or perhaps removed.  The metaphor of a pathway suggests a linear, unidirectional process of claim dissemination that is oversimplified. 
- Very minor: the authors state that they "randomly" selected one month from each quarter, but then say that they selected the second month of each quarter (which doesn't sound random).  This should be clarified.
- The authors make little of their finding, described in Figure 6, that maternal risk is far more frequently present in the media coverage than paternal risks.  What do the authors make of this result?
- The authors conclude that "risk messages relating to pregnancy and fertility are a common feature of UK print and online media."  This is an obvious enough conclusion, but to conclude it in this report would require some relative numbers.  Common relative to what other kinds of scientific risk messages, for example?

**Are sufficient details of methods and analysis provided to allow replication by others?**

 Yes, however,  the methods used to compare media reports and press releases to the original scientific articles could be elaborated.  Reframing this section as a narrative might be an effective way to address this issue, making clear that in this section observations are often mixed with evaluative language or commentary from the authors; that is is, it is a mix of reporting results and qualitatively discussing those results. 

**If applicable, is the statistical analysis and its interpretation appropriate?**

 Please explain why the columns in Figure 5 do not add to 56.

**Are all the source data underlying the results available to ensure full reproducibility?**

 Yes.

**Are the conclusions drawn adequately supported by the results?**

 Yes.

Is the work clearly and accurately presented and does it cite the current literature?

Yes

If applicable, is the statistical analysis and its interpretation appropriate?

Not applicable

Are all the source data underlying the results available to ensure full reproducibility?

Yes

Is the study design appropriate and is the work technically sound?

Yes

Are the conclusions drawn adequately supported by the results?

Yes

Are sufficient details of methods and analysis provided to allow replication by others?

Yes

Reviewer Expertise:

History and philosophy of science; gender studies of science.

I confirm that I have read this submission and believe that I have an appropriate level of expertise to confirm that it is of an acceptable scientific standard.

# Peer review: Reviewer response for version 1

**DOI:** 10.21956/wellcomeopenres.18465.r43901

**Published:** 2021-06-11

**Authors:** Norah MacKendrick

**Affiliations:** 1 Department of Sociology, Rutgers University, New Brunswick, NJ, USA

This is an important study that examines how the news media reports scientific research related to pregnancy and fertility. The research objectives are clear and the methodologies are appropriate. The manuscript would benefit from revisions, namely to fill in missing details across several sections of the paper.

**Introduction**

 The manuscript has two general objectives: assess i) risk framing in PRs and news media reports and, ii) the accuracy of news reporting of scientific studies related to fertility and pregnancy. The introduction could state more clearly these two general objectives. They appear only at the end of the introductory section (p. 3).

 In this section, the authors refer to the literature on the framing of risk messages in PRs and the impact of this framing on the public’s understanding of health risks and attribution of blame. These are important points that the authors re-visit in the discussion. The summary of this literature is very short and could be expanded upon. On page 3, for example, the authors cite several key articles (e.g. Reisch and Spiegelhalter; Tulloch and Zinn) but say little about the larger findings from this literature. This is important context that helps to set the stage for the study and the discussion section.

**Methods**

 The methods section is well organized and the methods used are appropriate. I appreciate the use of figures to show the reader the various stages of data collection and analysis. The authors could say more about why certain kinds of news articles were important and others were excluded. This section should also explain the rationale for the time period of 2018-19.

**Analysis**

 The analysis is detailed and the use of figures is helpful. The specific approach for evaluating accuracy remains vague. On pages 3-4, the authors refer to risk reporting pathways as a measure of reliability. If this is the method of measuring accuracy, the authors could tell us more about it.

 Figures 5 and 6 capture some elements of framing, as well as article content, but these figures could be explained in more detail in the text.

**Discussion**

 This section summarizes the main findings and their implications. The role of PRs in shaping media reports is an important finding. The authors could say even more about what they learned about the framing of pregnancy and fertility risks. Were risk messages in PRs the same as those in the media reports? What do we learn about risk framing in cases where the framing between PR and article diverged?

 Finally, the authors could revise the manuscript to add more text to the paper to guide the reader through each section of the manuscript. There are few transition and introductory sentences in each section, which gives the paper a “choppy” flow. Adding some of these would help the reader follow the paper from section to section.

 I appreciated the opportunity to review this paper and hope to cite it in my own work.

Is the work clearly and accurately presented and does it cite the current literature?

Yes

If applicable, is the statistical analysis and its interpretation appropriate?

Yes

Are all the source data underlying the results available to ensure full reproducibility?

Yes

Is the study design appropriate and is the work technically sound?

Yes

Are the conclusions drawn adequately supported by the results?

Yes

Are sufficient details of methods and analysis provided to allow replication by others?

Partly

Reviewer Expertise:

media framing, gender and risk, sociology of health, sociology of environmental risk

I confirm that I have read this submission and believe that I have an appropriate level of expertise to confirm that it is of an acceptable scientific standard.
